# Supplementary material for: Active learning regression quality prediction model and grinding mechanism for ceramic bearing grinding processing
Source: PLoS One. 2025 Apr 7;20(4):e0320494. doi: 10.1371/journal.pone.0320494 (PMC11975080; doi:10.1371/journal.pone.0320494)
Supplement: S1 Data — (DOCX) [file pone.0320494.s001.docx]

Figure 8 Model training loss values at different learning rates

| (a) Model training loss at a learning rate of 0.06 | | (b) Model training loss at a learning rate of 0.006 | | (c) Model training loss at a learning rate of 0.0006 | | (d) Model training loss at a learning rate of 0.00006 | |
| --- | --- | --- | --- | --- | --- | --- | --- |
| Iteration times/times | Model training loss value | Iteration times/times | Model training loss value | Iteration times/times | Model training loss value | Iteration times/times | Model training loss value |
| 0 | 0.11 | 0 | 0.07 | 0 | 0.040 | 0 | 0.080 |
| 500 | 0.03 | 500 | 0.01 | 500 | 0.001 | 500 | 0.001 |
| 1000 | 0.01 | 1000 | 0.01 | 1000 | 0.001 | 1000 | 0.001 |
| 1500 | 0.01 | 1500 | 0.01 | 1500 | 0.001 | 1500 | 0.001 |
| 2000 | 0.01 | 2000 | 0.01 | 2000 | 0.001 | 2000 | 0.001 |

Figure 9 Comparison of the performance curves of the two models

| (a) ROC curve | | | (b) PR curve | | |
| --- | --- | --- | --- | --- | --- |
| False Negative / % | Sensitivity / % (CNN-LSTM) | Sensitivity / % (AL-CLSTM) | Recall / % | Precision / % (CNN-LSTM) | Precision / % (AL-CLSTM) |
| 0 | 0.64 | 0.82 | 0 | 0.91 | 1.00 |
| 0.2 | 0.83 | 0.93 | 0.2 | 0.90 | 1.00 |
| 0.4 | 0.85 | 0.94 | 0.4 | 0.92 | 0.95 |
| 0.6 | 0.89 | 0.98 | 0.6 | 0.91 | 0.88 |
| 0.8 | 0.92 | 0.98 | 0.8 | 0.76 | 0.82 |
| 1.0 | 0.96 | 0.98 | 1.0 | 0.57 | 0.71 |

Figure 10 Comparison of model errors in different volume datasets—(a) CWRU Bearing Data Center

| Iteration | CNN-LSTM | DTRL | CNN-SBULSTM | AL-CLSTM |
| --- | --- | --- | --- | --- |
| 0 | 4.00 | 3.95 | 3.95 | 3.95 |
| 10 | 1.55 | 2.35 | 1.15 | 0.95 |
| 20 | 1.32 | 2.12 | 0.92 | 0.52 |
| 30 | 1.25 | 2.02 | 0.75 | 0.42 |
| 40 | 1.20 | 1.92 | 0.63 | 0.35 |
| 50 | 1.15 | 1.85 | 0.55 | 0.28 |
| 60 | 1.12 | 1.82 | 0.45 | 0.18 |
| 70 | 1.10 | 1.80 | 0.38 | 0.12 |

Figure 10 Comparison of model errors in different volume datasets—(b) Paderborn University Bearing Data Center

| Iteration | CNN-LSTM | DTRL | CNN-SBULSTM | AL-CLSTM |
| --- | --- | --- | --- | --- |
| 0 | 3.50 | 3.50 | 3.50 | 3.50 |
| 10 | 1.56 | 1.72 | 1.34 | 1.21 |
| 20 | 1.12 | 1.29 | 0.96 | 0.83 |
| 30 | 0.97 | 1.15 | 0.72 | 0.61 |
| 40 | 0.92 | 1.09 | 0.67 | 0.53 |
| 50 | 0.88 | 1.03 | 0.62 | 0.46 |
| 60 | 0.85 | 0.98 | 0.58 | 0.41 |
| 70 | 0.83 | 0.94 | 0.53 | 0.38 |
