# Supplementary material for: Active learning regression quality prediction model and grinding mechanism for ceramic bearing grinding processing
Source: PLoS One. 2025 Apr 7;20(4):e0320494. doi: 10.1371/journal.pone.0320494 (PMC11975080; doi:10.1371/journal.pone.0320494)
Supplement: Attachment — Submitted filename: Response to reviewers comments.docx [file pone.0320494.s003.docx]

Review Comments to the Author

Please use the space provided to explain your answers to the questions above. You may also include additional comments for the author, including concerns about dual publication, research ethics, or publication ethics. (Please upload your review as an attachment if it exceeds 20,000 characters)

Reviewer #1: 1. At the end of the Introduction section, write the scientific hypotheses.

Reply: Thank you for your suggestion. At the end of the introduction, the scientific hypothesis has been written as follows:

The study proposes the following hypothesis. Firstly, by combining active learning and deep learning models, the accuracy and reliability of surface quality prediction in ceramic bearing grinding can be significantly improved. Then, in the grinding process of ceramic bearings, optimizing grinding parameters (including wheel linear velocity, grinding depth, and feed rate) can significantly improve surface roughness, and the relative error range of the prediction model can be controlled within a small range. Finally, the optimization process based on active learning regression quality prediction model can effectively reduce the cost of experimental data annotation and improve the efficiency of grinding parameter optimization.

2.How are the parameters shown in table 1 selected?

Reply: Thank you for your comments. The study first conducted experimental analysis on the performance of the algorithm itself. Based on commonly used hyperparameter settings in relevant literature, key parameters such as learning rate, training epochs, and standardization parameters were preliminarily selected. Subsequently, these parameters were optimized using grid search methods to determine the optimal combination, ensuring the convergence of model training and the accuracy of predictions. In addition, hardware configuration is also selected based on experimental computing requirements to support efficient processing of large-scale data[31]. The experimental environment and related algorithm parameter settings are shown in Table 1.

[31] R. Naushad, T. Kaur, and E. Ghaderpour, "Deep transfer learning for land use and land cover classification: A comparative study," Sensors, vol. 21, no. 23, pp. 8083-8096, Dec. 2021. doi: 10.3390/s21238083.

3.How the selected grinding parameters are shown in table 4. (speed, depth and feed)? Why are they representative for your research?

Reply: Thank you for your comments. The reason for choosen these parameters has been explained as follows:

The linear speed of the grinding wheel, grinding depth, and feed rate are key factors affecting surface roughness. The linear velocity of the grinding wheel affects the contact frequency and cutting force between the abrasive particles and the workpiece, which in turn affects the grinding efficiency and temperature, while the grinding depth and feed rate directly affect the material removal rate and grinding force. The selection of these parameters is to explore their impact on surface roughness, in order to find a balance between improving grinding efficiency and ensuring quality.

Different parameters of ceramic grinding processing can have an impact on surface roughness. Firstly, the selection range for the linear velocity of the grinding wheel (unit: m/s) is 30 to 45 m/s. The grinding line speed has a significant impact on surface roughness, and higher line speeds usually reduce cutting forces, thereby reducing surface roughness. Secondly, the setting range of grinding depth (unit: mm) varies from 0.005 to 0.015 mm. The selection of this parameter is based on the characteristics of ceramic materials, and excessive grinding depth may lead to increased tool wear and surface damage. Finally, the selection range of feed rate (unit: mm/min) is 1000 to 5000 mm/min. The feed rate is directly related to the material removal rate and surface quality. A reasonable feed rate can improve production efficiency while ensuring the fineness of the processed surface. The experimental results are shown in Table 4.

4.What is the uncertainty of the obtained results?

Reply: Thank you for your review. The uncertainty of the obtained results has been stated in the discussion section as follows:

When conducting a systematic analysis of the uncertainty of the obtained results, it is considered that the uncertainty mainly comes from the accuracy of the measuring instruments, environmental changes, and differences in the characteristics of the materials themselves. To quantify these uncertainties, multiple repeated experiments were conducted and the standard deviation (SD) was calculated for each set of experimental data. Finally, the uncertainty of the surface roughness results evaluated using the uncertainty propagation formula was ± 0.003 μ m. This analysis provides an important basis for evaluating the reliability of experimental data.

5.In general, the results discussion must be enlarged and more comparisons with literature data should be reported. The authors should go more in depth with the interpretation of the results;

Reply: Thank you for your comments. The comparisons with literature data have been discussed in details and the interpretation of the results have been further explained as follows:

Comparing the experimental results of the study with relevant literature can not only provide a deeper understanding of the impact of grinding parameters on surface quality but also highlight the unique advantages of this study. For example, S. M. Lee's research suggests that the mechanism by which different abrasives affect surface roughness is closely related to their cutting performance and the dissipation of grinding heat. This indicates that under the same grinding conditions, the characteristics of the material determine the differences in machining performance. In this study, a step further is taken by precisely quantifying the surface roughness of ceramic materials within a specific range, fluctuating between 1.03% and 2.86%. This detailed quantification allows for a more accurate assessment of the material's behavior during grinding compared to previous general descriptions in the literature, and it is consistent with the description of the interaction between material removal and grinding in tribology. Meanwhile, the new grinding head developed by L. Jiang et al. significantly reduced surface roughness and grinding force, demonstrating the potential for improving tool design to achieve higher grinding quality. While that innovation focused on the tool design aspect, this study adopts a more comprehensive approach. Not only the impact of tool design is considered, but also a variety of grinding parameters and their interactions are systematically explored. Through a series of carefully designed experiments, the optimal combination of these parameters that maximizes the improvement in machining efficiency is identified, which is consistent with the goal of optimizing grinding parameters to improve machining efficiency in this study and provides a more holistic solution for achieving high grinding quality.

In the comparison of different grinding methods, X. Wang's surface finishing technology for precision bearing rolling elements emphasizes the balance between surface roughness and geometric errors during the grinding process. In contrast, this study not only supports the view that improving surface quality requires maintaining cost - effectiveness and production efficiency but also presents a novel methodology for evaluating and optimizing these aspects simultaneously. A unique evaluation model is developed that takes into account multiple factors specific to the experimental setup and application scenarios, enabling more targeted and practical recommendations for industrial applications.

Overall, by comparing with existing literature, it becomes evident that this study offers distinct contributions and advantages in furthering the understanding and improvement of grinding processes and surface quality.

6.Please write more theoretical support in the discussion section, so far only limited to stating the results. You need to extract valuable ideas from the data.

Reply: Thank you for your comments. More theoretical support has been provided in the discussion section, as follows:

In the grinding process of ceramic bearings, the influence of different grinding parameters on surface roughness is closely related to multiple factors, including grinding speed, grinding depth, and feed rate[32]. These factors are considered as key variables affecting the material removal mechanism in tribology theory[33]. The experimental results show that as the grinding speed increases, the surface roughness significantly decreases, indicating that optimizing the grinding speed plays a key role in improving the surface smoothness of ceramic materials, which is consistent with the theory of grinding force and contact state in tribology. Meanwhile, the combination of grinding depth and feed rate also has a significant impact on the generation of grinding force and heat. According to Hertz contact theory, as the grinding depth increases, the contact surface pressure also increases, which not only leads to the generation of more heat but also causes the failure of the grinding tool[34]. Therefore, appropriate selection of grinding depth can ensure machining quality and reduce tool wear. The setting of feed rate is directly related to the cutting quantity. For brittle ceramic materials, a lower feed rate helps to reduce instantaneous thermal shock and lower the risk of cracking during the machining process.

[32] K. Kishore, M. K. Sinha, A. Singh, Archana, M. K. Gupta, and M. E. Korkmaz, "A comprehensive review on the grinding process: advancements, applications and challenges," Proc. Inst. Mech. Eng., Part C: J. Mech. Eng. Sci., vol. 236, no. 22, pp. 10923-10952, Jun. 2022. doi: 10.1177/09544062221110782.

[33] B. Chen, L. Luo, H. Jiao, S. Li, S. Li, Z. Deng, and H. Yao, "Affecting factors, optimization, and suppression of grinding marks: A review," Int. J. Adv. Manuf. Technol., vol. 115, no. 1, pp. 1-29, Jul. 2021. doi: 10.1007/s00170-021-07116-0.

[34] Y. Liu, S. Song, G. Xiao, Y. Huang, and K. Zhou, "A high-precision prediction model for surface topography of abrasive belt grinding considering elastic contact," Int. J. Adv. Manuf. Technol., vol. 125, no. 1, pp. 777-792, Mar. 2023. doi: 10.1007/s00170-022-10757-4.

Reviewer #2: The reviewer suggests that the manuscript can be accepted after the author resolves the conflicting parts, clarifies the ambiguous parts and corrects the formatting issues.

1.On page 9, in lines 4 and 15, “cBN” should be changed to “CBN”.

Reply: Thank you for your review. "cBN" has been changed to "CBN" .

2.On page 10, in the ninth line from the bottom, the sequence of “cutting, plowing, and friction” should be changed to “scraping, plowing, cutting”.

Reply: According to your kind suggestions, "cutting, plowing, and friction" has been revised to "scraping, plowing, cutting".

3.On page 11, Figure 1 does not effectively demonstrate the three stages of abrasive cutting, and Figure 2 should be centered.

Reply: Thank you for your feedback. Figure 2 has been centered; The three stages of abrasive cutting have been supplemented in Figure 1, as follows:

Figure 1. Three stages of abrasive cutting process

4.On page 12, both Formula 1 and Formula 2 represent the calculation of the critical values for the generation of radial cracks, and further elaboration is needed.

Reply: Thank you for your suggestion. The difference between Formula 1 and Formula 2 has been explained as follows:

Both formulas are used to calculate the critical value for radial crack generation, but they consider different factors and focus. Formula 1 is more directly related to the fracture toughness and hardness of materials, while Formula 2 further considers the ratio of elastic modulus to hardness, which has a more complex impact on the formation of cracks in materials.

5.On page 19, the conclusion in the seventh line differs from what is depicted in Figure 8(b). In the tenth line, "a range of 0.10 to 0.10" should be corrected as it represents a single value rather than a range, and this should be revised in conjunction with Figure 8(c). Additionally, the legend in Figure 9(c) does not correspond with the colors in the image.

Reply: Thank you for your comments. The description of Figure 8 (b) has been corrected. And the error in the description of Figure 8 (c) has been corrected as follows:

**The description of Figure 8 (b) has been corrected:**

Figure 8 (b) illustrates the trend of the model training loss value when LR is 0.006. HA is still the number of iterations, and VA represents the loss value. The loss value starts at approximately 0.07 and gradually decreases to around 0.001 as the number of iterations increases.

**The error in the description of Figure 8 (c) has been corrected as follows:**

The loss value starts at 0.80 and gradually decreases to a range of 0.00 to 0.10 as the iterations proceed.

**The legend and color in Figure 9 (c) have been modified as follows:**

**Figure 9. Comparison of the performance curves of the two models**

6.It is recommended that each value in the "Average training loss" column should be written on a separate line instead of spanning two lines.

Reply: Thank you for your review. Each value in the "Average training loss" column has been written on a separate row, as follows:

**Table 2. Experimental results of AL-CLSTM for ceramic bearing grinding processing**

| Model | Iteration count | Learning rate | Average training loss | Average prediction error | Convergence time (s) | Accuracy (%) |
| --- | --- | --- | --- | --- | --- | --- |
| AL-CLSTM | 2000 | 0.06 | 0.03572 | 0.04589 | 250 | 94.32 |
| CNN-LSTM | 2000 | 0.006 | 0.02714 | 0.03854 | 280 | 92.17 |
| CNN-SBLSTM | 2000 | 0.0006 | 0.04122 | 0.05211 | 320 | 90.45 |
| DTRL | 2000 | 0.00006 | 0.06509 | 0.08072 | 450 | 86.90 |
| Baseline model (linear regression) | 2000 | N/A | 0.08945 | 0.10234 | 180 | 82.50 |
| CNN-RNN | 2500 | 0.002 | 0.03418 | 0.04132 | 350 | 91.80 |
| SAX-Predict | 1500 | 0.004 | 0.04734 | 0.05648 | 310 | 88.25 |

7.On page 23, the two graphs in Figure 11 are of different sizes and need to be adjusted to match. This suggests that for consistency and clarity, the figures should be resized so that they are of equal dimensions within the figure.

Reply: Thank you for your review. The size of the graph in Figure 11 has been adjusted to have equal dimensions within the graph, as follows:

**Figure 11. Ceramic bearings**

8.In the conclusion section of the article, examples are needed to illustrate the situations in which the accessibility of data may be restricted.

Reply: Thank you for your suggestion. Examples of situations where data acquisition may be limited have been provided in the conclusion, as follows:

Although this study has drawn meaningful conclusions, data acquisition may be limited by factors such as sample size, degree of control over the experimental environment, and variables that may not have been fully considered during the grinding process, including tool wear status, material changes during the grinding process, fluctuations in grinding force due to machine vibration, and the influence of coolant properties on the grinding interface. These limitations may affect the accuracy of experimental results. So in future studies, the sample size and number of experimental replicates should be increased, and the impact of various factors, such as those mentioned above, on the results should be further explored to improve the comprehensiveness and credibility of the research.

Reviewer #3: The manuscript focuses on predicting the quality of ceramic bearings during grinding. The manuscript presents an active learning regression model for the construction and optimization of a roughness model. The variable parameters are linear grinding wheel speed, grinding depth and feed rate. The conducted experiments showed that the mentioned parameters affect the surface roughness of the workpiece.

The presented experimental data are useful for practical applications. However, the scientific value of the regression model needs to be adjusted. For this purpose, it is necessary to answer the following principal comments.

1.The influence of grinding modes on surface roughness is known. The authors have not found any differences for grinding ceramic bearings.

Reply: Thank you for your suggestion. The differences in grinding ceramic bearings have been added to the introduction as follows:

In particular, L. D. M. Fernandes et al. discovered that ceramics exhibit excellent mechanical properties under high - temperature, high - speed, and high - load conditions. This makes them ideal for bearing applications, yet also poses unique challenges during grinding[2]. However, U. Patel and V. Patel pointed out that traditional grinding processing (GP) often fails to meet the surface roughness (SR) requirements when grinding high - hardness ceramic bearings. This not only leads to subpar bearing performance but also highlights the significant difference between grinding ceramic bearings and other materials. Ceramic bearings demand higher precision in surface finish due to their application in high - performance equipment, and traditional GP struggles to deliver[3]. Electrolytic in - process dressing (ELID) grinding shows promise for optimizing grinding quality and efficiency. But as D. Xiang et al. and B. Dou et al. revealed, most existing studies mainly focus on single grinding parameters. They lack a comprehensive exploration of the multilevel effects of material properties and grinding conditions specific to ceramic bearings. For instance, the unique hardness and brittleness of ceramic materials during bearing grinding can result in different chip formation and heat dissipation patterns compared to other materials. These differences require a more in - depth understanding of the grinding mechanism and better - tailored quality prediction models[4-5].

The process of grinding ceramic bearings is significantly different from grinding materials such as metals or plastics. Ceramic materials typically have extremely high hardness and low toughness, which makes them prone to brittle fracture and surface cracks during grinding. In contrast, metal materials are more prone to plastic deformation during grinding, and their stress distribution is relatively uniform. In addition, the thermal sensitivity of ceramic materials makes them more sensitive to the heat generated during grinding, and excessively high temperatures may lead to a decrease in material properties and deterioration of surface quality. Therefore, the study of grinding parameters and methods for ceramic bearings is particularly crucial to prevent the generation of cracks and ensure higher machining quality.

2.There is no physical explanation for the results obtained.

Reply: Thank you for your review. Physical explanations have been added to the results section as follows:

When the learning rate is too low, the step size of parameter updates becomes smaller, causing the model to wander around the optimal solution of the loss function, and the convergence process appears very slow.

When AL-CLSTM has 3000 iterations and a learning rate of 0.03, it still exhibits better average training loss and prediction error. This indicates that even with a decrease in learning rate, the model can still maintain good convergence and accuracy.

When the grinding depth is 20 μ m, the average training loss of the AL-CLSTM model is 0.04237, and the average prediction error is 0.05312, indicating that this setting can basically achieve good prediction performance. However, when the grinding depth increased to 21 μ m, the average training loss of the model further decreased to 0.03622, and the surface roughness Ra value also significantly decreased to 0.1624 μ m. This change can be explained by the stress distribution and heat generation mechanism during the grinding process. A larger grinding depth increases the cutting area, allowing the cutting force and friction force during the cutting process to be fully utilized, which helps improve the efficiency of material removal and reduce the local heat generated during the grinding process, thereby reducing microcracks caused by thermal stress. Meanwhile, the increase in grinding speed (from 40m/s to 45m/s) also had a positive impact on the prediction accuracy of the model. During the grinding process, a higher linear velocity of the grinding wheel can reduce the contact time between the material and the grinding wheel, decrease the accumulation of frictional heat, and mitigate its adverse effects on surface quality. Meanwhile, the increase in grinding speed (from 40m/s to 45m/s) also had a positive impact on the prediction accuracy of the model. During the grinding process, a higher linear velocity of the grinding wheel can reduce the contact time between the material and the grinding wheel, decrease the accumulation of frictional heat, and mitigate its adverse effects on surface quality.

3.The results for roughness reduction are close to the measurement error.

Reply: Thank you for your review. The shortcomings of the roughness reduction results approaching the measurement error have been added in the conclusion section, as follows:

Although the study observed an improvement in surface roughness, the variation in roughness was only 0.001 to 0.002 μ m due to measurement errors (e.g. ± 0.003 μ m). This means that the observed improvement may not exceed the range of measurement error. Therefore, in further research, it is necessary to optimize the measurement process to reduce experimental errors and ensure accurate reflection of the impact of grinding parameter optimization on surface quality. Meanwhile, based on statistical analysis, as the sample size increases, it is expected to obtain more significant results to verify the true impact of grinding operations on the surface quality of ceramic bearings.

4.When grinding ceramic bearings, significantly lower roughness values than Ra = 0.2 µm are achievable.

Reply: Thank you for your review. A new round of experiments has been conducted to achieve roughness values lower than Ra=0.2 µ m. The data in Table 4 has been updated as follows:

**Table 4. Experimental results of the influence of different parameters on surface roughness in ceramic grinding processing**

| Experiment number | Wheel linear speed (m/s) | Grinding depth (mm) | Feed rate (mm/min) | Surface roughness Ra (μm) | Reverse grinding Ra (μm) | Relative error (%) |
| --- | --- | --- | --- | --- | --- | --- |
| 1 | 30 | 0.008 | 1000 | 0.2012 | 0.2228 | 2.78 |
| 2 | 35 | 0.010 | 2000 | 0.1964 | 0.2157 | 1.83 |
| 3 | 40 | 0.012 | 3000 | 0.1908 | 0.2089 | 1.37 |
| 4 | 45 | 0.015 | 4000 | 0.1876 | 0.2063 | 0.95 |
| 5 | 32 | 0.005 | 1500 | 0.1943 | 0.2246 | 2.12 |
| 6 | 38 | 0.011 | 2500 | 0.1917 | 0.2118 | 1.74 |
| 7 | 42 | 0.009 | 3500 | 0.1849 | 0.2034 | 1.05 |
| 8 | 34 | 0.014 | 5000 | 0.1894 | 0.2121 | 1.13 |

Table 4 summarizes the experimental results of the influence of different grinding parameters on the surface roughness of ceramic grinding. With the increase of grinding wheel linear velocity and moderate adjustment of grinding depth, the AL-CLSTM model exhibits lower surface roughness Ra values under various experimental conditions. For example, when the grinding wheel linear velocity is set to 45 m/s and the grinding depth is 0.015 mm, the Ra value drops to 0.1876 μ m, indicating that the optimization of grinding parameters significantly improves the machining quality. Grinding force, heat generation, and material properties are the main physical factors that affect the results. Moderate grinding depth can reduce cutting force and tool damage, while high linear velocity effectively reduces cutting time, reduces heat accumulation, and avoids cracks in ceramic materials caused by overheating during processing. In addition, the high hardness and brittleness of ceramic materials require stress control during grinding to prevent crack propagation. Therefore, the reasonable selection of grinding wheel line speed and feed rate is the key to improving surface smoothness.

5.It is not sufficient to evaluate the quality of machining by one roughness parameter alone.

Reply: Thank you for your review. More experimental parameters have been added to evaluate the quality of machining, as follows:

The experimental results of the influence of grinding wheel material particle size, lubricant composition, and processing time on the surface roughness of ceramic bearings are shown in Table 5.

**Table 5 Impact of Different Parameters on Ceramic Bearing Surface Roughness**

| Experiment No. | Wheel Material | Grain Size (μm) | Lubricating Fluid Composition | Grinding Time (min) | Surface Roughness Ra (μm) | Relative Error (%) |
| --- | --- | --- | --- | --- | --- | --- |
| 1 | CBN | 100 | Water-based | 15 | 0.2104 | 1.85 |
| 2 | CBN | 150 | Water-based | 15 | 0.1987 | 1.72 |
| 3 | CBN | 200 | Oil-based | 20 | 0.1873 | 1.66 |
| 4 | CBN | 300 | Oil-based | 20 | 0.1861 | 1.50 |
| 5 | Diamond | 100 | Water-based | 10 | 0.2022 | 1.80 |
| 6 | Diamond | 150 | Oil-based | 25 | 0.1829 | 1.40 |
| 7 | Diamond | 200 | Oil-based | 25 | 0.1725 | 1.20 |
| 8 | CBN | 250 | Water-based | 30 | 0.1974 | 1.80 |

The experimental results in Table 5 indicate that as the grain size of the grinding wheel increases, the surface roughness usually shows a decreasing trend. When using a 100 μ m CBN grinding wheel and water-based lubricant, the surface roughness reaches 0.2104 μ m, while when using a 150 μ m CBN grinding wheel, the surface roughness decreases to 0.1987 μ m. Although in the experiment with a grinding time of 15 minutes, the application of oil-based lubricant achieved better results of 0.1873 μ m at a particle size of 200 μ m, indicating a significant impact of lubricant composition on the grinding effect. Overall, during the grinding process, the smaller the particle size of the grinding wheel, combined with appropriate lubricant composition and processing time, can more effectively reduce surface roughness and improve the overall processing quality of ceramic bearings.

6.The surface roughness during grinding is more influenced by the grain size of the grinding wheel material, the composition of the lubricating fluid, and the machining time. These factors were not considered in the model.

Reply: Thank you for your review. The experimental results on the influence of grinding wheel material particle size, lubricant composition, and processing time on the surface roughness of ceramic bearings have been supplemented in Table 5.

7.Data on grinding machine, grinding wheel, measuring devices are not given.

Reply: Thank you for your suggestion. The data for the grinder, grinding wheel, and measuring device have been provided as follows:

The grinding machine used in the experiment is the YE7232 model from Qinchuan Machine Tool Group Co., Ltd. It is equipped with a 400mm diameter grinding wheel made of cubic boron nitride (CBN) with a particle size of 100 #. We chose this type of grinding wheel because it exhibits excellent grinding efficiency and surface quality when grinding ceramic materials. In addition, the measurement of surface roughness was carried out using the JB-1C model measuring equipment from Shanghai Precision Instrument Co., Ltd., which has high precision and repeatability, ensuring the reliability and accuracy of the data.
